# Retraction Note to: WRN inhibits oxidative stress-induced apoptosis of human lensepithelial cells through ATM/p53 signaling pathway and its expression is downregulated by DNA methylation

**DOI:** 10.1186/s10020-021-00417-w

**Published:** 2021-12-07

**Authors:** Shengqun Jiang, Jiansu Chen

**Affiliations:** 1 Ophthalmology Department, The First Affiliated Hospital of Jinan University Guangzhou, No.601 Huangpu Avenue West, Guangzhou, Guangdong Province China; 2 Ophthalmology Department, The First Affiliated Hospital of Bengbu Medical College, Bengbu, Anhui Province China

## Retraction to: Mol Med (2020) 26:68 10.1186/s10020-020-00187-x

The authors have retracted this article because after publication they became aware that the concentrations of H_2_O_2_ used to treat the SRA01/04 cells in this study had been miscalculated. In addition, the experiment reported in Fig. 2d did not have a negative control. The results and conclusions presented in this article are therefore unreliable. All authors agree to this retraction.

